# Hospital Notes

**Published:** 1919-06

**Authors:** 


### HOSPITAL NOTES.

The Registered Nurses Association of Dallas gave an informal reception in the Baptist Sanitarium in honor of the Red Cross Nurses who have returned from the service.

The Alpine Hospital has been re-opened by Dr. J. R. Middle-Brook. Katherine Solcum and Louise Satterlee, Red Cross Army Nurses who were recently discharged from Fort Sam Houston, are the nurses in charge.

The Baylor Hospital Unit organized in the fall of 1917 and sent to Europe in June 1918, after service upon the European battlefields, has demobilized.

Citizens of Orange, Texas, are making an effort to have the Emergency Hospital in that city continued as a city institution.

Miss Gertrude Shaffer, formerly of Brenham, has resigned her position as assistant superintendent of nurses at a San Antonio hospital to accept a position as superintendent of a hospital at Kerrville.

Charles Hodgon, of Chicago, conferring with the Board of Directors of Baylor Hospital College said the chief need of North Texas is a hospital to meet the demands of the outlying country about Dallas. He said that there ought to be one bed in the hospitals to every one hundred people in the country and that in hospital facilities Dallas and North Texas are far behind the requirements.

Most of the drink cure institutions are preparing to discontinue business with the coming of national prohibition, according to officials of such institutions in Chicago. They expect business to increase for a few months right after prohibition becomes effective but to decrease rapidly after that.

With the settlement of certain complications arising from the ministering certain conditions of the will of the late George H. Hermann the building programme for the prospective Hermann Hospital at Houston is taking definite shape.

General Merritt W. Ireland of the United States Army says that there will be a sustained need of 2,250 nurses in the permanent army establishments of the Red Cross.

The Cty Commission of Fort Worth is contemplating the erecof the largest institutions of its kind in the South, as a fitting memorial to Tarrant County boys who lost their lives in the service.

